# Evaluation of X Chromosome Inactivation with Respect to HLA Genetic Susceptibility in Rheumatoid Arthritis and Systemic Sclerosis

**DOI:** 10.1371/journal.pone.0158550

**Published:** 2016-06-29

**Authors:** Sami B. Kanaan, Onur E. Onat, Nathalie Balandraud, Gabriel V. Martin, J. Lee Nelson, Doua F. Azzouz, Isabelle Auger, Fanny Arnoux, Marielle Martin, Jean Roudier, Tayfun Ozcelik, Nathalie C. Lambert

**Affiliations:** 1 Institut National de la Santé et de la Recherche Médicale (INSERM) UMRs1097, Scientific Park of Luminy, Marseille, France; 2 Aix-Marseille University, Marseille, France; 3 Department of Molecular Biology and Genetics, Bilkent University, Ankara, Turkey; 4 Service de Rhumatologie, Hôpital Sainte Marguerite, AP-HM, Marseille, France; 5 Clinical Research Division, Fred Hutchinson Cancer Research Center, Seattle, Washington, United States of America; 6 Division of Rheumatology, University of Washington, Seattle, Washington, United States of America; Medical University of South Carolina, UNITED STATES

## Abstract

**Background:**

Autoimmune diseases, including rheumatoid arthritis (RA) and systemic sclerosis (SSc) are characterized by a strong genetic susceptibility from the Human Leucocyte Antigen (HLA) locus. Additionally, disorders of epigenetic processes, in particular non-random X chromosome inactivation (XCI), have been reported in many female-predominant autoimmune diseases. Here we test the hypothesis that women with RA or SSc who are strongly genetically predisposed are less susceptible to XCI bias.

**Methods:**

Using methylation sensitive genotyping of the androgen receptor (*AR*) gene, XCI profiles were performed in peripheral blood mononuclear cells from 161 women with RA, 96 women with SSc and 100 healthy women. *HLA-DRB1* and *DQB1* were genotyped. Presence of specific autoantibodies was documented for patients. XCI skewing was defined as having a ratio ≥ 80:20 of cells inactivating the same X chromosome.

**Results:**

110 women with RA, 68 women with SSc, and 69 controls were informative for the *AR* polymorphism. Among them 40.9% of RA patients and 36.8% of SSc patients had skewed XCI compared to 17.4% of healthy women (*P* = 0.002 and 0.018, respectively). Presence of RA-susceptibility alleles coding for the “shared epitope” correlated with higher skewing among RA patients (*P* = 0.002) and such correlation was not observed in other women, healthy or with SSc. Presence of SSc-susceptibility alleles did not correlate with XCI patterns among SSc patients.

**Conclusion:**

Data demonstrate XCI skewing in both RA and SSc compared to healthy women. Unexpectedly, skewed XCI occurs more often in women with RA carrying the shared epitope, which usually reflects severe disease. This reinforces the view that loss of mosaicism in peripheral blood may be a consequence of chronic autoimmunity.

## Introduction

Female predominance in autoimmune diseases is remarkable as approximately 80% of patients are women [1, 2]. Rheumatoid arthritis (RA), and systemic sclerosis (SSc) are both examples of autoimmune diseases that follow this rule with women:men ratios going from 3:1 in RA to 11:1 in SSc [2]. RA and SSc are often characterized by the presence of autoantibodies in patients’ sera. The most specific autoantibodies in SSc are antitopoisomerase antibodies (ATA) and anticentromere antibodies (ACA), which are respectively a hallmark of diffuse cutaneous SSc and limited cutaneous SSc; the two clinical subtypes of the disease [3]. Autoantibodies encountered in RA are the rheumatoid factor (RF) and the highly specific anti-citrullinated protein antibodies (ACPA) [4], and they can precede the clinical manifestation of RA by many years [5, 6].

As in most autoimmune diseases, gene polymorphisms in the Human Leucocyte Antigen (HLA) locus account for the highest genetic risk in the development of RA and SSc. In RA, several *HLA-DRB1* alleles (*01:*01*, *01:*02*, *01:*04*, *04:*01*, *04:*04*, *04:*05*, *04:*08*, *10:*01* and *14:*02*) are implicated [7]. They code for a shared motif of five amino-acid sequence ^70^Q(or R)-K(or R)-R-A-A^74^, the so-called *shared epitope* (SE), in the third hypervariable region of the DRβ1 molecule [8]. A parallel to the SE of RA can be made in SSc. Indeed, disease subtypes and autoantibody profiles are strongly associated with *HLA-DRB* and *DQB* alleles, such as DRB1*11, *08 (in European and African-American subjects) DRB5*01:*01* (in linkage disequilibrium with the Asian susceptibility allele DRB1*15:*02*), DQB1*03, *06, etc. [9–11]. Most *DRB* alleles associated with SSc have in common an amino-acid sequence ^67^F-L-E-D-R^71^ on their β chain. Similarly, most *DQB* susceptibility alleles code for a common ^71^T-R-A-E-L-D-T^77^ motif on their β chain, and both motifs are often associated with an ATA producing SSc profile [11, 12].

It is likely that X-linked risk factors have a role to play in disease onset and progression. Skewed X chromosome inactivation (XCI) is well established in peripheral blood cells from women with autoimmune thyroiditis and SSc [13–16]. Only one report shows such a bias in RA from a North African population [17] and one in juvenile idiopathic arthritis [18].

XCI is an epigenetic dosage compensation mechanism in female mammalian cells where either the paternally-derived or the maternally-derived X chromosome is randomly silenced in early embryonic life [19]. Skewing then represents a deviation from the 50:50 ratio and is arbitrarily defined, often as a pattern were 80% or more (≥ 80:20) of the cells inactivate the same X chromosome [20]. This deviation is thought to be the result of i) genetic factors directly involved in the process of XCI, ii) genetic defects (mutations, rearrangements,…) on the X chromosome leading to a selective process, iii) tendency towards monoclonal expansion of cells related to aging, or iv) pure chance, due to the stochastic nature of the choice of which X chromosome to inactivate in the early stages of embryogenesis [21, 22].

The most accepted explanation to biased XCI in autoimmunity is a mechanism through which loss of mosaicism has the potential to make X-linked self-antigens escape presentation in the thymus, leading to the breakdown of tolerance and causing the development of autoimmune diseases [23]. Although this view lacks supporting evidence and remains to be confirmed, one would expect that women with autoimmune disease who are strongly genetically predisposed are less susceptible to skewed XCI. This is also reinforced by the observation that HLA genes’ contribution to the RA or SSc risk is substantially greater in men than in women [24, 25]. Indeed, men do not have the epigenetic possibility of biased XCI and are more genetically predisposed than women, when affected [24, 25]. We then hypothesize that women with RA and SSc would have less skewed XCI patterns if carrying one of the strongest risk predictors in both diseases: susceptibility in the HLA class II locus.

## Methods

### Participants’ characteristics

Among the 357 female subjects included in the study, a total of 110 women with RA (median age and interquartile range (IQR): 58 [51–66] years), 68 women with SSc (55 [48–54] years), and 69 healthy women (52 [46–58] years) were informative for the XCI assay and were considered in subsequent analyses. Among the 69 control women, 65 were Caucasian (94.2%), 3 from Sub-Saharan African Ancestry, and 1 Asian. Patients with RA were recruited from the rheumatology service of Marseille’s hospitals (France) and satisfied the 2010 revised criteria of the American College of Rheumatology and the European League Against Rheumatism [26]. Of them, 108 were Caucasian (98.2%), 1 African, and 1 Asian. Patients with SSc were enrolled in collaboration with 5 French hospitals from Paris, Marseille and Lille, and met the requirements of LeRoy [27]. Among them, 55 were Caucasian (80.8%), 8 were African, and 5 Asian. Median age, with IQR, at the onset of disease was 48 [41–57] and 46 [37–55] years, and median disease duration was 6 [1–17] and 6 [4–12] years, for RA and SSc respectively. The selection criterion for healthy controls was no symptoms or familial history of autoimmune disorder.

To evaluate XCI patterns and the SE in autoimmune disease other than RA, women with SSc were tested. As women with SSc who carry the RA-specific SE are rare, we added women with SSc previously described in a North American study, who had comparable XCI pattern with our French SSc cohort to increase statistical power [16]. Among the 94 additional SSc women informative in the XCI assay, *HLA-DRB1* and *DQB1* genotyping was retrospectively obtained for 92 of them.

Positivity for ACPA was used to define mainstream RA because of its well-recognized diagnostic and predictive value [4]. Therefore, all selected patients with RA were ACPA-positive. Positivity for ACPA was obtained from patients’ clinical files for 17 out of 110 RA patients. For the remaining 93 patients, ACPA were detected by anti-CCP2 Enzyme-linked immuno-sorbent assay (ELISA) (Immunoscan RA, Euro-Diagnostica, Arnhem, The Netherlands). Positivity was defined by a cut-off value of 25 Units/mL at a dilution of 1:50 of patients’ plasma. Median ACPA titration, with IQR, was 528 [228–896] Units/mL.

For the French patients with SSc, autoantibody profiles (ATA, ACA) and patients’ disease subtypes were obtained by reviewing medical records. Among them, 37 had the diffuse cutaneous form of the disease, with 24 of them being positive for ATA, and 31 had the limited cutaneous form, with 2 of them being positive for ATA and 20 for ACA. Thirty-six patients were assessed for anti-RNA Pol III autoantibodies by Quanta Lite anti-RNAP III ELISA kit (INOVA Diagnostics, San Diego, USA) following manufacturer’s recommendations, only one was positive (3%). Clinical characteristics and methods of DNA extraction and HLA genotyping for the American SSc patients were described elsewhere [28].

### Ethics statement

The study has received the approval of the ethics committee (*Comité de Protection des Personnes Sud-Méditerranée II*, *CPP*) and is registered at the INSERM under the Biomedical Research Protocol number RBM-04-10 or as a collection under the number DC-2008-327. All participants signed informed consent according to the Declaration of Helsinki [29].

### PBMC and plasma isolation, and DNA extraction

Peripheral blood mononuclear cells (PBMCs) and plasma were isolated from 8 mL of whole blood (drawn in EDTA or heparin vacutainer tubes) using Ficoll Histopaque 1077 (Sigma-Aldrich, St Louis, MO, USA) gradient centrifugation. Plasma was stored at −80°C. Genomic DNA was extracted from PBMCs with EZ1 DNA Tissue Kit (Qiagen, Hilden, Germany) using a BioRobot EZ1 system (Qiagen, Hilden, Germany) according to the manufacturer’s instructions, and stored at −20°C.

### *HLA-DRB1* and *HLA-DQB1* genotyping

*HLA-DRB1* typing was carried out for all subjects at the Etablissement Français du Sang (Marseille, France) and/or in our laboratory, using sequence-specific oligonucleotide (SSO) typing kits (Dynal, Invitrogen, Carlsbad, CA, USA; SSO LABType, One Lambda Inc., CA, USA) for generic typing and/or sequence-specific primer (SSP) typing kits (Olerup HLA-DRB1*04, Genovision, Vienna, Austria) for allelic typing. *HLA-DQB1* typing was done for all subjects except RA patients. SSO typing kits were used for this purpose (RELI SSO, Dynal, Invitrogen, Bromborough, Wirral, UK).

### X chromosome inactivation assay

**Fig 1:**
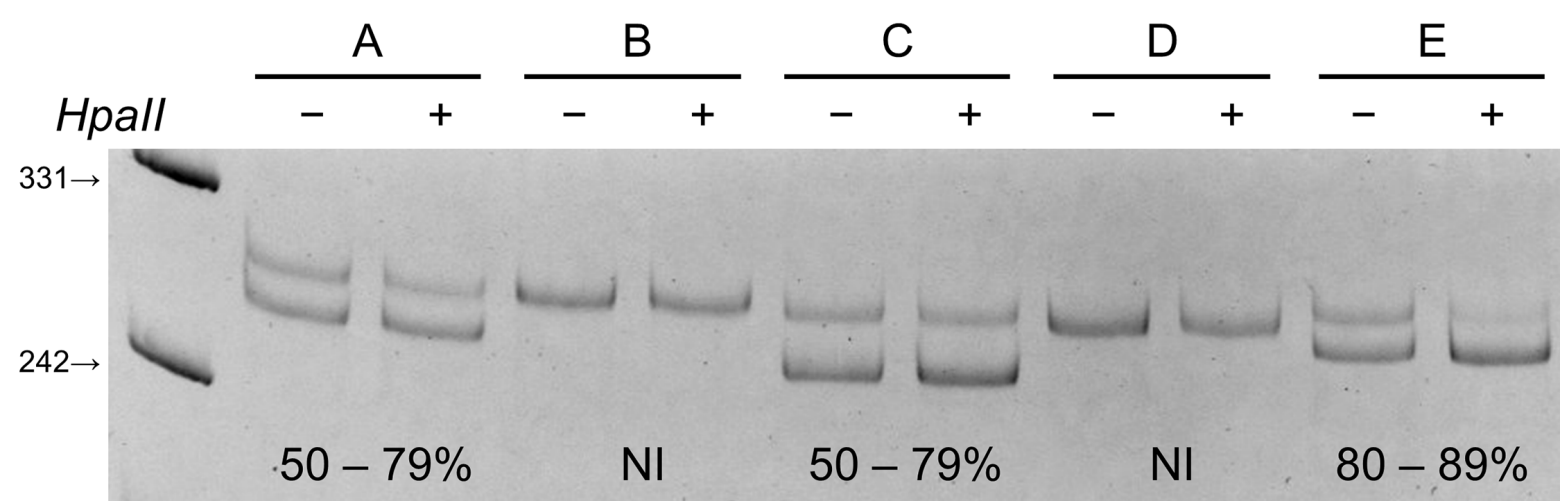
Example of X chromosome inactivation status in 5 samples. Polymerase chain reaction products from the androgen receptor (*AR*) methylation assay shows random X chromosome inactivation (50–79%) in samples A and C, and skewed X chromosome inactivation (80–89%) in sample E. Samples B and D have a non-informative status. For each sample, DNA was either undigested (−) or digested (+) with the methylation-sensitive restriction enzyme *HpaII*. Marker (331-bp and 242-bp) fragments are visible.

Genotyping of a polymorphic site in the human androgen receptor (*AR*) gene was performed and quantified based on the use of radioactive α-^33^P-dCTP (NEN, Boston, MA, USA) to assess the XCI patterns as previously described [13, 30]. DNA methylation occurs on the inactive X, and prevents a methylation-sensitive *HpaII* restriction enzyme to cleave on its specific site located on exon 1 of *AR* gene. When the genomic DNA is cleaved with *HpaII* prior to PCR, only the methylated *AR* allele, which represents the inactive X-chromosome, is amplified. A polymorphic CAG repeat located within the amplified region is used to distinguish between the two alleles (Fig 1). For each patient and control the same polymerase chain reactions (PCRs) was performed on two samples, with or without *Hpa*II treatment. Male DNA with verified 46XY karyotype was used as control for complete digestion. Densitometric analysis of the alleles was performed at least twice for each sample using the MultiAnalyst version 1.1 software (Bio-rad, Hercules, California, USA). A corrected ratio was calculated by dividing the ratio of the predigested sample (upper/lower allele) by the ratio of the non-predigested sample for normalization of the ratios that were obtained from the densitometric analyses. The use of corrected ratio compensates for preferential amplification of the shorter allele when the number of PCR cycles increases [31]. A skewed population is defined when a cell population represents 80% or higher of one of the *AR* alleles (mosaicism ratio of ≥ 80:20) and extremely skewed when mosaicism is at a ratio ≥ 90:10. It is noted that French and American patients with SSc have been tested for XCI with the same methods.

### Statistical analyses

A categorical approach was chosen to statistically analyze XCI data, in consistency with the historical way of classifying XCI patterns into random, skewed, and extremely skewed. An *r* × *c* Fisher’s exact test (*r* and *c* represent respectively a number of rows and a number of columns) was applied in contingency analyses. This test gives exact *P* values and is particularly robust when expected values in contingency tables are sparse [32]. When expected values were large (≥ 5), the difference between exact and approximate *P* values would be minor and either Fisher’s exact or chi-square “*Χ*^2^” test were used (with Yates’ correction in case of 2 × 2 contingency tables).

A continuous approach was also applied through the non-parametric two-sample Kolmogorov–Smirnov (K–S) test. K–S is notably useful to test the equality of continuous probability distribution, and is sensitive to differences in both location and shape of the cumulative distribution functions of two compared samples. Exact *P* values in K–S were computed to better account for smaller sample sizes. Continuous correlation was assessed with the non-parametric Spearman rank test. *P* values less than 0.05 were considered significant. Statistical analyses were conducted using GraphPad Prism 5 software (La Jolla, CA, USA) STATA 14 (College Station, TX, USA), and the online tool “Statistics to Use” (Kirkman, T.W. http://www.physics.csbsju.edu/stats/).

## Results

### Women with RA and women with SSc have more often a skewed XCI pattern

Individuals for whom paternally-derived and maternally-derived *AR* gene alleles could not be distinguished were not included in the analysis and considered as non-informative. XCI status was informative for 110 of the 161 RA patients (68.3%), 69 of the 100 healthy women (69.0%), and 68 of the 96 SSc patients (70.8%) from the present study, comparable to the reported 75.2% in the North American SSc patients [16].

Overall, XCI patterns significantly differed between patients and healthy controls. Skewed patterns (≥ 80:20) were more frequent among women with RA or women with SSc when compared to healthy women (respectively, 40.9% [45/110], 36.8% [25/68] versus 17.4% [12/69]). *P* values were respectively 0.002 and 0.018 (*Χ*^2^ with Yates’ correction). This result would remain significant with a Bonferroni correction (two comparisons).

**Fig 2:**
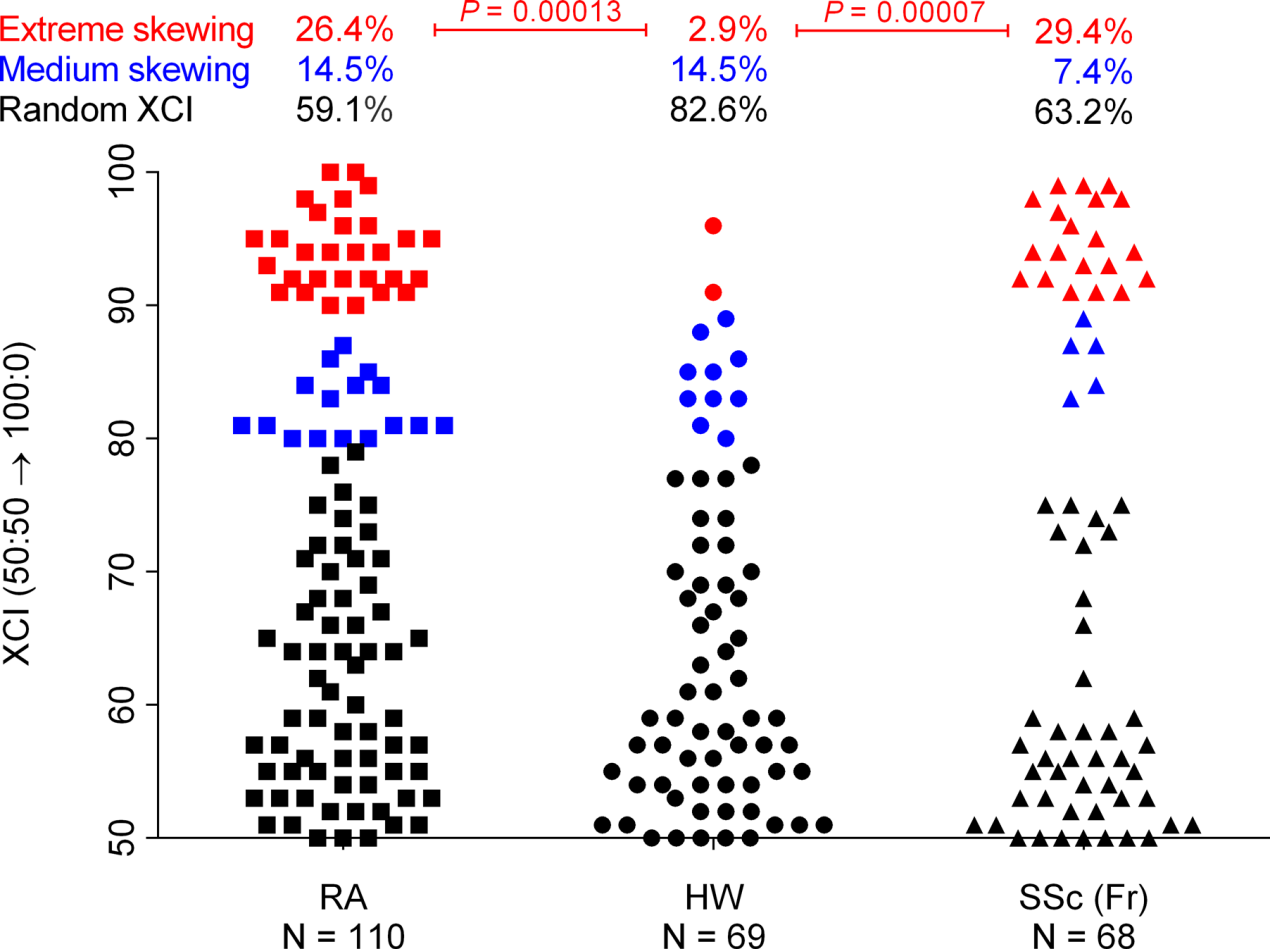
Degrees of X chromosome inactivation (XCI) in healthy women (HW), women with rheumatoid arthritis (RA), and women with systemic sclerosis (SSc) in the French cohort (Fr). Upper part: Proportional analysis with respect to the three categories of XCI: Extreme skewing (90:10 → 100:0), Medium skewing (80:20 → 89:11), and Random (50:50 → 79:21). *P* values were from *Χ*^2^ with Yates’ correction. Lower part: Scatter plots showing a difference of the distribution of XCI patterns in women with RA and SSc compared to HW (*P* = 0.013 and 0.009 respectively, Kolmogorov–Smirnov).

Statistical differences were even more pronounced when comparing frequencies of extremely skewed XCI patterns (≥ 90:10) between patients and controls. Indeed, 26.4% (29/110) of RA women, 29.4% (20/68) of French SSc women had an extremely skewed pattern compared to only 2.9% (2/69) among healthy women (*P* = 0.00013 and 0.00007 respectively; *Χ*^2^ with Yates’ correction; Fig 2).

When modeling XCI patterns through a continuous approach, probabilities of random XCI patterns decreased and those of skewed XCI patterns increased significantly in women with RA and SSc compared to healthy women (*P* = 0.013 and 0.009 respectively, K–S).

No statistical difference was observed in XCI patterns between RA and SSc patients (*P* = 0.35; *Χ*^2^ test and *P* = 0.15, K–S).

### Skewed XCI occurs more often with the presence of the SE among women with RA

XCI patterns were evaluated with respect to the presence of genetic susceptibility in the HLA locus. In the context of RA, the presence of *HLA-DRB1* alleles coding for the SE motif was analyzed in association with XCI patterns. Of the 247 women informative for XCI status, 235 were *HLA-DRB1* genotyped at the allelic level. The presence of the SE, as expected, was more frequent among women with RA. Indeed, SE was present in double dose (SE+/+) in 20.8% (21/101) and in single dose (SE+/−) in 51.5% (52/101) of RA patients, compared to only 7.2% (5/69) and 27.6% (19/69) respectively in healthy women, and only 3.1% (2/65) and 20% (13/65) respectively in SSc patients from the present study.

The prevalence of a skewed XCI was associated with the presence of the SE in women with RA. The contingency matrix showed that skewed patterns were significantly more common among women with RA when they had SE-positive alleles (*r* × *c* Fisher’s exact test, *P* = 0.002; Table 1). Probability distribution of XCI patterns in women with RA revealed the most skewing when they had a double dose of SE compared to both a single dose of SE or none (*P* = 0.045 and 0.008; K–S). This effect was not apparent for RA women with one dose of SE compared to no SE (*P* = 0.294, K–S). On the other hand, with either categorical or continuous statistics, no significant association of XCI patterns could be observed with the presence of the SE among healthy women or among women with SSc, whether from our French cohort or from the North American one. These two groups of SSc women were combined to increase statistical power in the analysis and represented in Table 1. Such combination seemed appropriate after verifying that XCI pattern did not differ between the French and American women with SSc (*P* = 0.10, *Χ*^2^ test), as random, skewed, and extremely skewed patterns were present in 62 (66.0%), 15 (16.0%), and 17 (18.1%) of the 94 American patients assayed for XCI.

**Table 1:** Comparison of X chromosome inactivation patterns and the presence of HLA-susceptibility alleles.

	N	Random XCI (50:50 79:21)	Medium skewing (80:20 89:11)	Extreme skewing (90:10 100:0)	*P* value^†^
SE* **in RA**					
−/−	28	21 (75.0%)	4 (14.3%)	3 (10.7%)	
+/−	52	29 (55.8%)	11 (21.1%)	12 (23.1%)	***0*.*002***
+/+	21	9 (42.9%)	0 (0.0%)	12 (57.1%)	
SE* **in HW**					
−/−	45	38 (84.5%)	6 (13.3%)	1 (2.2%)	
+/−	19	15 (78.9%)	4 (21.1%)	0 (0.0%)	*0*.*29*
+/+	5	4 (80.0%)	0 (0.0%)	1 (20.0%)	
SE* **in SSc**^‡^					
−/−	106	68 (64.2%)	11 (10.4%)	27 (25.5%)	
+/−	42	27 (64.3%)	8 (19.0%)	7 (16.7%)	*0*.*56*
+/+	9	6 (66.7%)	1 (11.1%)	2 (22.2%)	
^**67**^**FLEDR**^**71**^ **in SSc**^‡^					
−/−	59	38 (64.4%)	10 (16.9%)	11 (18.6%)	
+/−	72	45 (62.5%)	9 (12.5%)	18 (25.0%)	*0*.*49*
+/+	27	19 (70.4%)	1 (3.7%)	7 (25.9%)	
^**71**^**TRAELDT**^**77**^ **in SSc**^‡^					
−/−	25	16 (64.0%)	3 (12.0%)	6 (24.0%)	
+/−	74	44 (59.5%)	11 (14.9%)	19 (25.7%)	*0*.*74*
+/+	59	42 (71.2%)	6 (10.2%)	11 (18.6%)	

*SE represents the ^70^Q(R)K(R)RAA^74^ motif carried by “shared epitope”-positive *HLA-DRB1* alleles that predispose to RA, but not to SSc. Therefore, SSc-associated shared amino-acid motifs ^67^FLEDR^71^ (from some *DRB1* and *DRB5* alleles) and ^71^TRAELDT^77^ (from some *DQB1* alleles) were considered as additional controls in this context.

^†^*r* × *c* Fisher’s exact test.

^‡^Women with SSc described in this analysis are compiled results from French women and North American women. HW: healthy women; RA: women with rheumatoid arthritis; SE: shared epitope; SSc: women with systemic sclerosis; XCI: X chromosome inactivation.

Susceptibility alleles specific to SSc were analyzed in comparison to XCI profiles. HLA-DR and DQ genotyping was available for 158 of the 162 informative French and American SSc patients to identify FLEDR-positive *DRB1* and *DRB5* alleles and TRAELDT-positive *DQB1* alleles. No statistically significant association of their presence with XCI skewing could be observed in the French SSc group, or the North American group (data not shown) or both combined (Table 1).

### XCI patterns are not associated with age, disease duration, disease subtype, treatments or presence of autoantibodies

In addition to genetic susceptibility, we proposed to analyze whether presence of autoantibodies in plasma of patients, age, disease duration, subtype of disease (in the case of SSc) were correlated with XCI patterns. French and North American patients with SSc had similar XCI profiles, and they were combined in Table 2 to increase statistical power.

**Table 2:** Contingency analyses of X chromosome inactivation patterns in regard to various subjects’ characteristics.

	N	Random XCI (50:50 79:21)	Medium skewing (80:20 89:11)	Extreme skewing (90:10 100:0)	*P* value^†^
***Healthy women***					
Age (years)*					
36–46	21	17 (81.0%)	4 (19.0%)	0 (0.0%)	
47–57	28	22 (78.6%)	5 (17.8%)	1 (3.6%)	*0*.*56*
58–69	20	18 (90.0%)	1 (5.0%)	1 (5.0%)	
***RA women***					
Age (years)*					
27–44	17	12 (70.6%)	1 (5.9%)	4 (23.5%)	
45–62	51	34 (66.7%)	7 (13.7%)	10 (19.6%)	*0*.*34*
63–80	36	17 (47.2%)	7 (19.5%)	12 (33.3%)	
**Disease duration (years)**					
0–9	63	40 (63.5%)	12 (19.0%)	11 (17.5%)	
10–19	21	14 (66.7%)	1 (4.8%)	6 (28.5%)	*0*.*12*
≥ 20	24	11 (45.8%)	3 (12.5%)	10 (41.7%)	
**ACPA titers (U/mL)**					
25–75 (low-positive)	9	7 (77.8%)	0 (0.0%)	2 (22.2%)	
≥ 76 (high-positive)	84	47 (55.9%)	13 (15.5%)	24 (28.6%)	*0*.*43*
***SSc women***^‡^					
Age (years)*					
18–38	31	23 (74.2%)	4 (12.9%)	4 (12.9%)	
39–59	91	56 (61.5%)	13 (14.3%)	22 (24.2%)	*0*.*51*
60–80	39	25 (64.1%)	3 (7.7%)	11 (28.2%)	
**Disease duration (years)**					
0–9	129	83 (64.3%)	16 (12.4%)	30 (23.3%)	
10–19	20	11 (55.0%)	4 (20.0%)	5 (25.0%)	*0*.*50*
≥ 20	12	10 (83.3%)	0 (0.0%)	2 (16.7%)	
**Disease type**					
Limited cutaneous	58	40 (69.0%)	6 (10.3%)	12 (20.7%)	
Diffuse cutaneous	101	64 (63.4%)	12 (11.9%)	25 (24.8%)	*0*.*82*
**ATA**^§^					
Positive	50	27 (54.0%)	7 (14.0%)	16 (32.0%)	
Negative	89	63 (70.8%)	9 (10.1%)	17 (19.1%)	*0*.*14*^*¶*^
**ACA**^§^					
Positive	28	17 (60.7%)	5 (17.9%)	6 (21.4%)	
Negative	95	63 (66.3%)	7 (7.4%)	25 (26.3%)	*0*.*28*

*Range of years was equally divided by 3, accordingly forming 3 groups.

^†^*r* × *c* Fisher’s exact test.

^‡^SSc women from both current and American studies are represented here in the analyses.

^§^ATA and ACA information were missing from 23 and 39 cases out of the 94 SSc from the American study.

^¶^When analyzing ATA-positive versus negative and random XCI versus skewed XCI (≥ 80:20) in a 2 × 2 approach, *P* value was 0.06 (Fisher’s exact) or 0.08 (Kolmogorov–Smirnov’s exact) for continuous XCI distribution. ACA: anticentromere antibody; ACPA: anti-citrullinated protein antibody; ATA: antitopoisomerase antibody; RA: rheumatoid arthritis; SSc: systemic sclerosis; XCI: X chromosome inactivation.

Despite this, the subset analysis in Table 2 was susceptible to be underpowered, as sample sizes tended to be small on many occasions. Age did not appear to have an effect on XCI in all groups, and neither disease duration in women with RA and SSc. A correlation analysis revealed Spearman R coefficients all between −0.02 and 0.11 (data not shown). XCI patterns were not significantly associated (Fisher and K–S tests) with low or high ACPA titers among women with RA, where high-positive ACPA was defined by a value superior to three times the upper limit of a normal titer [26], or 3 × 25 Units/mL in this study. XCI patterns were not significantly associated (Fisher and K–S tests) with a limited or diffuse cutaneous form of SSc, and presence of ACA among women with SSc. Women with SSc positive for ATA had a tendency to have more often a skewed XCI pattern, without reaching significance (*P* = 0.08, K–S test; *P* = 0.06, 2 × 2 Fisher’s exact test). These negative results held for SSc whether analyzed from our current study, replicated in the American study (data not shown), or both combined (Table 2).

Women with RA who had skewed XCI had similar treatments as patients with random XCI. Among patients with RA for whom we had treatment information, 64% of patients with biased XCI (N = 28) were under anti-TNF treatments (etanercept and infliximab) versus 62% of patients with unbiased XCI (N = 34). Other treatments were only methotrexate or only corticoids or both combined and were similarly found in both groups (respectively 36% and 38%).

## Discussion

Many studies have shown an association between skewed XCI and autoimmunity [13–18], but none had analyzed any correlation with genetic susceptibility, or other variables that characterize diseases. We show a higher prevalence of skewed XCI in peripheral blood cells of women affected with RA and SSc compared to healthy women, in agreement with those previous studies. This prevalence is particularly remarkable when comparing extreme skewing in RA and SSc patients versus controls. Furthermore, XCI patterns found in our group of healthy women match those described in large cohorts of controls [33], supporting the idea that they correspond to patterns in the general population.

Next, we found that women with RA were likelier to have skewed patterns when they carried SE-positive alleles (*P* = 0.002). This result goes in the opposite direction of our initial hypothesis, stating that if genetic and epigenetic factors both contribute to disease susceptibility, one would be more present when the other is lacking. Importantly, this association between SE and skewing was not noticed among control women, whether healthy or with SSc. Contrary to what was found in RA, analysis of SSc-specific HLA-susceptibility alleles, namely the FLEDR-positive and TRAELDT-positive alleles, with skewed patterns among women with SSc suggested that HLA predisposition in SSc and X chromosome mosaicism were unrelated.

Although we used Fisher’s exact test that gives exact *P* values, robust when numbers in contingency tables are sparse, and computed exact *P* values when K–S test was used, we remained limited in group sizes. As expected, there were few healthy women with extreme skewing (N = 2) or healthy women and women with SSc who carry a double dose of the RA-specific SE (N = 5 and 2, respectively). For better statistical power, we were able to combine our group of women with SSc with a North American group previously described for XCI but never tested for HLA association with skewing [16]. Whether both SSc groups were analyzed together or separately, the *HLA-DRB1* and *DQB1* susceptibility alleles remained not associated with XCI patterns and, importantly, women with SSc who carried the RA-specific SE were not preferentially skewed. This reinforces our observation that the influence of RA-specific HLA-susceptibility alleles on XCI patterns is limited to RA, and similar influence is not seen in SSc even with SSc-specific HLA-susceptibility alleles.

A skewed XCI has been proposed as an X-linked risk factor in the development of autoimmunity [13–18]. In this perspective, our findings may suggest that skewed inactivation of the X chromosome and polymorphisms of HLA genes on chromosome 6 are synergic risk factors in RA. If this is true however, a skewed XCI would also be more prevalent in subjects carrying the SE, even without RA. This assumption remains unlikely, as it does not appear to be the case in our group of healthy controls or in the group of women with another autoimmune condition, i.e. SSc, for which the SE does not confer susceptibility.

Our data demonstrate a correlation between skewing and the presence of the SE, the main genetic susceptibility marker associated with RA pathogenesis. The SE is also considered a marker of severe disease activity, cartilage erosion and bone destruction, regardless of therapy [34–36]. Accordingly, a model can be proposed where skewed XCI is a consequence of the severity of the autoimmune condition, a result of an accelerated rate of turnover in selected clones of immune cells. One can suspect the involvement of CD4^+^ T cells and antigen presenting cells, on the grounds that the SE is part of HLA class II molecules. Interestingly, XCI bias has been previously shown associated with a deregulated Foxp3 expression, an X-linked molecule, on CD4^+^ T regulatory cells from patients with SSc [37]. As a result of this proposed model, skewing is expected to increase with disease duration. A tendency towards more frequent XCI skewing already appears in our data among RA patients with the longest disease duration, although statistically not significant (*P* = 0.12, Table 2). In SSc on the other hand, disease severity often depends on the clinical subtypes and, ultimately, is associated with autoantibody profiles [3, 38]. Of the two classical autoantibodies in SSc, ATA predict severe outcomes and higher mortality rates [3, 38]. In this case also, XCI bias is expected to occur more often in SSc patients when ATA are present. Our data show a tendency towards more frequent skewing among ATA-positive SSc patients, without being statistically significant. It is possible that tendencies not reaching statistical significance are the consequence of lack of power, due to smaller sample sizes in the sub-groups analyses of Table 2. Our proposed model thus merits further confirmation on larger RA and SSc cohorts.

In summary, the present study demonstrates the association of biased XCI patterns with RA and SSc in a French population, comes in agreement with previous findings on XCI in autoimmunity, and presents the first evidence of a disease-specific association between loss of mosaicism and HLA susceptibility (in RA). In this direction, data are robust, dually controlled against both healthy individuals and individuals with another autoimmune disease. The association of a skewed pattern with the presence of the SE in RA is less in favor of skewing as a contributor to autoimmunity. Rather, skewing as a consequence of cell division in chronic disease seems to be the most plausible explanation accounting for our findings. Next steps should address a number of important questions, including the immuno-phenotype of cells most likely to express skewed patterns, whether certain X-linked polymorphisms contribute to clonal-expansion-derived XCI bias, and to which extent the autoimmune environment influences such bias.
